# Insights into divalent cation regulation and G_13_-coupling of orphan receptor GPR35

**DOI:** 10.1038/s41421-022-00499-8

**Published:** 2022-12-21

**Authors:** Jia Duan, Qiufeng Liu, Qingning Yuan, Yujie Ji, Shengnan Zhu, Yangxia Tan, Xinheng He, Youwei Xu, Jingjing Shi, Xi Cheng, Hualiang Jiang, H. Eric Xu, Yi Jiang

**Affiliations:** 1 CAS Key Laboratory of Receptor Research, Center for Structure and Function of Drug Targets, Shanghai Institute of Materia Medica, Chinese Academy of Sciences, Shanghai, China; 2 University of Chinese Academy of Sciences, Beijing, China; 3 School of Pharmacy, Macau University of Science and Technology, Macau, China; 4 School of Pharmaceutical Science and Technology, Hangzhou Institute of Advanced Study, Hangzhou, Zhejiang China; 5 School of Life Science and Technology, ShanghaiTech University, Shanghai, China; 6 State Key Laboratory of Drug Research, Shanghai Institute of Materia Medica, Chinese Academy of Sciences, Shanghai, China; 7 Lingang Laboratory, Shanghai, China

**Keywords:** Cryoelectron microscopy, Hormone receptors

## Abstract

Endogenous ions play important roles in the function and pharmacology of G protein-coupled receptors (GPCRs) with limited atomic evidence. In addition, compared with G protein subtypes G_s_, G_i/o_, and G_q/11_, insufficient structural evidence is accessible to understand the coupling mechanism of G_12/13_ protein by GPCRs. Orphan receptor GPR35, which is predominantly expressed in the gastrointestinal tract and is closely related to inflammatory bowel diseases (IBDs), stands out as a prototypical receptor for investigating ionic modulation and G_13_ coupling. Here we report a cryo-electron microscopy structure of G_13_-coupled GPR35 bound to an anti-allergic drug, lodoxamide. This structure reveals a novel divalent cation coordination site and a unique ionic regulatory mode of GPR35 and also presents a highly positively charged binding pocket and the complementary electrostatic ligand recognition mode, which explain the promiscuity of acidic ligand binding by GPR35. Structural comparison of the GPR35–G_13_ complex with other G protein subtypes-coupled GPCRs reveals a notable movement of the C-terminus of α5 helix of the Gα_13_ subunit towards the receptor core and the least outward displacement of the cytoplasmic end of GPR35 TM6. A featured ‘methionine pocket’ contributes to the G_13_ coupling by GPR35. Together, our findings provide a structural basis for divalent cation modulation, ligand recognition, and subsequent G_13_ protein coupling of GPR35 and offer a new opportunity for designing GPR35-targeted drugs for the treatment of IBDs.

## Introduction

Cations are highly abundant in the biological system and play an essential role in the regulation of G protein-coupled receptors (GPCRs). Na^+^ has been found to stabilize the inactivate conformational state and allosterically inhibit GPCR activation^1^. The Na^+^-binding site, denoted by the sodium pocket, has been proven conserved by a number of GPCR structures^2–5^. Besides Na^+^, other cations are also involved in the allosteric modulation of GPCRs. Ca^2+^ enhances the ligand activity by structurally engaging agonist and melanocortin receptors MC1R^6^ and MC4R^7^. Mg^2+^ is also known to promote agonist binding to the μ-opioid receptor^8,9^ and oxytocin receptor (OTR)^10^, serving as a positive allosteric modulator. However, the binding sites of diverse divalent cations and the underlying allosteric mechanisms are still far from fully understood.

G protein heterotrimers, comprising α, β, and γ subunits, are recognized and activated by agonist-bound GPCRs. At least 18 different Gα subunits are encoded in mammals, which can be grouped into four subfamilies, including G_s_, G_i/o_, G_q/11_, and G_12/13_. Upon coupling to agonist-bound GPCRs, the G_12/13_ subfamily activates the Ras-superfamily small G protein Rho A, regulating numerous physiological functions, including cell growth, differentiation, and actin cytoskeletal reorganization^11,12^, and is involved in pathology processes, such as cardiovascular disorders, metabolic diseases, and cancer^12–15^. Compared to other G protein subtypes, engineered G_13_-coupled GPCR structures have not been reported until recently^16,17^. The scarcity in GPCR–G_12/13_ complex structures constrains our comprehensive understanding of GPCR–G_12/13_ coupling. GPR35 is a representative GPCR coupled with G_13_^18^ and G_i/o_ proteins^19^, providing an opportunity to explore the molecular basis for G_13_ recruitment.

The orphan receptor GPR35 belongs to class A GPCR, first identified two decades ago, and remained poorly characterized due to a lack of pharmacological tools to probe its physiology and pharmacology. It was nominally deorphanized due to the finding that the tryptophan metabolic kynurenic acid modestly activates the receptor. It has been classified as metabolite-sensing GPCRs activated by endogenous metabolites, such as phospholipid derivate lysophosphatidic acid, chemokine CXCL17, and 5-hydroxyindoleacetic acid^20^. However, GPR35 is still officially defined as an orphan receptor as its exact endogenous activator has not been convincingly defined. GPR35 also responds to several clinical drugs, including anti-allergic mast cell stabilizers lodoxamide, bufrolin, and antinociceptive pamoic acid with low nanomolar potency^21^.

Human GPR35 is mainly expressed in the gastrointestinal (GI) tract, predominantly in the stomach, intestinal epithelial cells, dendritic cells, and macrophages of the small intestine and colon^22^. It plays a critical role in regulating GI homeostasis and provides an important link between metabolic, immune, and gut microbiota systems^23^. The inadequate GPR35 signaling is closely associated with an increased risk of inflammatory bowel diseases (IBDs), both ulcerative colitis and Crohn’s diseases, as well as primary sclerosing cholangitis^24–26^. Several coding variants, such as hyperactive T108M mutation in GPR35, increased the risk for IBDs^27,28^. Thus, GPR35 has attracted increasing interest as a drug target for IBD treatment. Interestingly, diarrhea, the most common symptom of IBDs, results, at least in part, from the intestinal hydroelectrolytic imbalance^29,30^. This disequilibrium is caused by absorptive ion transport and secretion defects, thus creating a probable linking across GPR35, ion homeostasis, and IBD pathogenesis. Although substantial progress has been made, the structural basis for recognition by a broad spectrum of ligands is unclear, and the insights into GPR35 activity regulation by ions and IBD-associated mutation remain to be elucidated. Here we determined the cryo-electron microscopy (cryo-EM) structure of the G_13_-coupled GPR35 bound to an anti-allergic drug, lodoxamide. Notably, our work identifies a novel divalent cation coordinate site and a unique allosteric agonism mode and also provides insights into ligand binding, receptor activation, and the G_13_ coupling of GPR35.

## Results

### Structure of GPR35

**Fig. 1:**
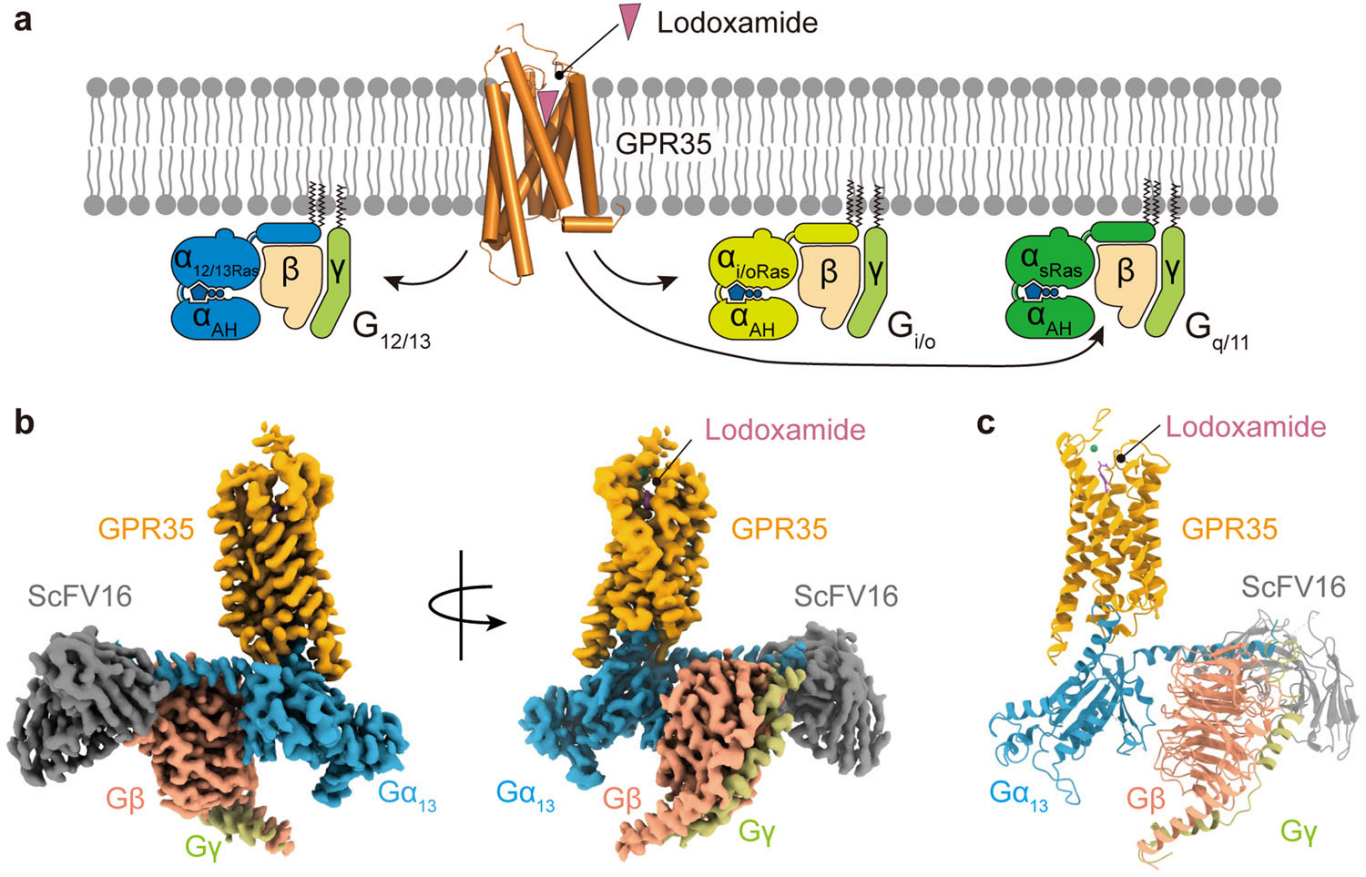
Overall structure of the lodoxamide–GPR35–G_13_–scFv16 complex. **a** Schematic diagram of lodoxamide-mediated activation and G protein coupling of GPR35. **b** Orthogonal views of the density map for the lodoxamide–GPR35–G_13_–scFv16 complex. GPR35 is shown in orange, Gα_13_ in slate blue, Gβ in salmon, Gγ in lime, scFv16 in grey, and lodoxamide in magenta. **c** Orthogonal views of the model of the lodoxamide–GPR35–G_13_–scFv16 complex.

We solved the structure of full-length human GPR35 in complex with a G_13_ heterotrimer, scFv16, and lodoxamide using cryo-EM analysis. The G_13_ heterotrimer used in our study contained an engineered Gα_13_ subunit, in which the N-terminus of wild-type Gα_13_ was replaced by that of Gα_i1_ to facilitate the binding of scFv16^31^, and was designated as Gα_13/iN_. Unless otherwise specified, G_13_ refers to this engineered G_13/iN_. The NanoBiT strategy^32^ was applied to facilitate GPR35-G_13_ assembly, with the LgBiT and HiBiT linking to the C-terminus of the receptor and Gβ subunit, respectively. The lodoxamide–GPR35–G_13_–scFv16 complex was assembled by co-expressing the receptor-LgBiT with Gα_13_, Gβ-HiBiT, Gγ subunits, and scFv16 in the presence of lodoxamide (Supplementary Fig. S1a). The final structure was determined at a resolution of 3.2 Å (Fig. 1; Supplementary Fig. S1 and Table S1). The cryo-EM map is sufficiently clear to fit the receptor, G_13_ protein heterotrimer, and the ligand into the complex. The overall structure of the complex consists of a canonical transmembrane domain of seven transmembrane helices (TM1–TM7), three extracellular loops (ECL1–ECL3), three intracellular loops (ICL1–ICL3), and an amphipathic helix, H8 (Supplementary Fig. S2). The high-quality map allowed accurate model building for receptor residues N6^NT^–A292^8.59^ (Fig. 1), which provides detailed atomic information of the ligand-binding pocket and the receptor–G_13_ protein coupling interface.

### Positive allosteric modulation of GPR35 by selected divalent cations

**Fig. 2:**
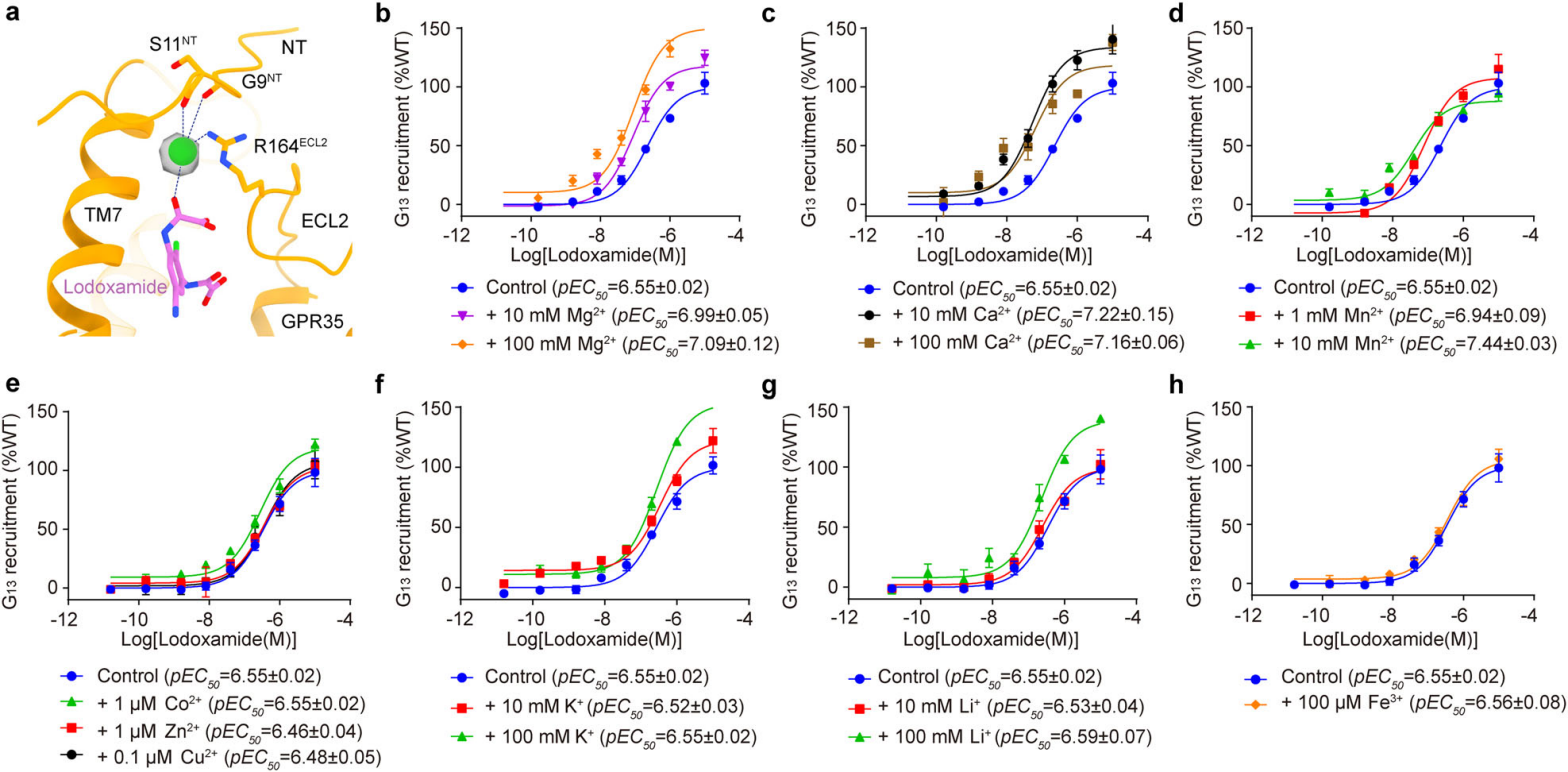
Allosteric agonism of GPR35 by cations. **a** Architecture of the cation coordination site. The cation is coordinated by the backbone oxygen of G9^NT^ and S11^NT^, the side chain of R164^ECL2^, and a carbonyl oxygen atom of lodoxamide. Cation in GPR35 is displayed as a green sphere, while its EM density is colored in grey. Interactions between the cation and surrounding residue coordinates are indicated as blue dashed lines. **b**–**h** Allosteric regulatory effects of different cations on lodoxamide-induced G_13_ recruitment by GPR35, including Mg^2+^ (**b**), Ca^2+^ (**c**), Mn^2+^ (**d**), Co^2+^, Zn^2+^, Cu^2+^ (**e**), K^+^ (**f**), Li^+^ (**g**), and Fe^3+^ (**h**). The concentrations of different cations are indicated. The physiological concentrations of Co^2+^, Zn^2+^, Cu^2+^, and Fe^3+^ are much lower than those of Mg^2+^ and Ca^2+^ (WHO Vitamin and Mineral Nutrition Information System, VMNIS). The maximum cell safety concentrations of these cations under our experimental conditions were used.

The most notable observation in the EM map of the lodoxamide–GPR35–G_13_–scFv16 complex is a strong spheroid density, which is embraced by the receptor N-terminus, ECL2, TM7, and the agonist lodoxamide (Fig. 2a). Considering its similar extracellular location with other cations in GPCRs and the relevance of cations in regulating GPCR^3,6,7,33^, we speculated that the undefined EM density map corresponds to a cation.

To identify the presence of a cation and the putative cation subtypes, we set up the NanoBiT G protein recruitment assay to assess the potential role of cations in allosteric agonism of exogenous agonists for GPR35. Under our experimental conditions, Mg^2+^ and Ca^2+^ substantially upregulated lodoxamide activity (Fig. 2b, c; Supplementary Table S2). Mn^2+^ made a comparable impact relative to Mg^2+^ (Fig. 2d), which is not surprising as the chemical and biochemical behavior of Mn^2+^ resembles that of Mg^2+^ and often replaces Mg^2+^ in the active site of a magnesium-utilizing enzyme^34^. A similar positive allosteric effect of Mg^2+^, Ca^2+^, and Mn^2+^ was also observed for zaprinast, a cGMP-PDE inhibitor that is an activator of GPR35 (Supplementary Fig. S3a). The allosteric regulation of Mg^2+^ and Ca^2+^ is consistent with a previous finding that the presence of 10 mM Ca^2+^ or 10 mM Mg^2+^ markedly promoted the binding of a radiolabeled GPR35 agonist^35^. However, these divalent cations failed to allosterically activate 5-HT_1A_ in the presence of endogenous agonist 5-HT. It should be noted that concentrations of divalent cations applied in our in vitro assay are ~1–5-fold and ~4–40-fold higher than the physiological concentrations of extracellular Mg^2+^ and Ca^2+^, respectively (vs 1.2–1.4 mM for Mg^2+^^36^ and 2.2–2.6 mM for Ca^2+^^37^). However, considering that ~300–400 mg Mg^2+^ and ~1000 mg of Ca^2+^ are taken up daily for a healthy adult, these high local concentrations can be achieved in the intestine, where GPR35 is abundantly distributed, and absorption of cations occurs. Intriguingly, GPR35 is not susceptible to other tested divalent cations, such as Co^2+^, Zn^2+^, and Cu^2+^, monovalent ions Li^+^, K^+^, and trivalent ion Fe^3+^ (Fig. 2e–h), in their maximum cell safety concentrations under our experimental conditions. These data corroborate the idea that specific divalent cations promote the positive allostery of GPR35, although the exact divalent cation subtype remains to be defined. The relative promiscuity of divalent cations for GPR35 is different from the high cation selectivity observed in reported GPCRs, such as Mg^2+^ for OTR^38^ and Ca^2+^ for MC1R^6^ and MC4R^7^, thus presenting a unique cation regulation mode of GPR35.

We further questioned whether divalent cations can be coordinated without an exogenous ligand. Besides the allosteric agonism effect, divalent cations Mg^2+^, Ca^2+^, and Mn^2+^ enhanced the G_13_ protein-coupling efficacy of apo GPR35 in a concentration-dependent manner (Supplementary Fig. S3b, c). These findings are consistent with the previous molecular simulation model that Mg^2+^ and Ca^2+^ positively propagate GPCR allosteric signals by bridging acidic residues within either ECL2 or ECL3 to lower the activation barrier between active and intermediate receptor states^39^. Similarly, 10 mM or even higher concentrations of monovalent cations Li^+^ and K^+^ showed detectable increases in G_13_ protein recruitment by GPR35 (Supplementary Fig. S3d). By contrast, divalent cations Zn^2+^, Co^2+^, Cu^2+^, and trivalent cation Fe^3+^ hampered G protein recruitment of GPR35 at a micromolar concentration (Supplementary Fig. S3e–g).

The structural comparison reveals a distinct cation-binding site in GPR35 in contrast to other cation-bound class A GPCRs. The location of the cation EM density in GPR35 approaches the extracellular receptor components but differs from the conserved sodium binding pocket buried in the TMD helices (Supplementary Fig. S4a). In addition, the cation in GPR35 shows a distinct extracellular location and engages with distinct receptor components compared with other reported divalent cation sites, including the Mg^2+^ site in OTR (PDB: 7RYC) and Ca^2+^ sites in MC1R (PDB: 7F4H) and MC4R (PDB: 6W25) (Supplementary Fig. S4b–e). Moreover, unlike the favorable binding mode of a divalent cation, which is coordinated by the side chains of the acidic/amide-containing residues, the cation in GPR35 forms interatomic interacts with the backbone oxygen of G9^NT^ (4.3 Å) and S11^NT^ (4.4 Å), the side chain of R164^ECL2^ (3.5 Å), and a carbonyl oxygen atom of lodoxamide (4.3 Å) (Supplementary Fig. S4). Since the typical Mg^2+^ and Ca^2+^ coordination distances are 2.07–2.29 Å and 2.37–2.49 Å^40^, respectively, solvent water may participate in these interactions. The lacking of strong electron donors surrounding divalent cations may explain the relatively weak allosteric agonism of divalent cations to GPR35 compared to OTR, MC1R, and MC4R (10 mM vs 0.5–2 mM of effective concentrations)^6,7,38^. Together, these findings provide insights into the positive allosteric modulation of GPR35 by Mg^2+^ and Ca^2+^. The structure of GPR35 is also added to the pool to enhance the understanding of the ionic regulation of GPCR.

### Ligand-binding pocket

**Fig. 3:**
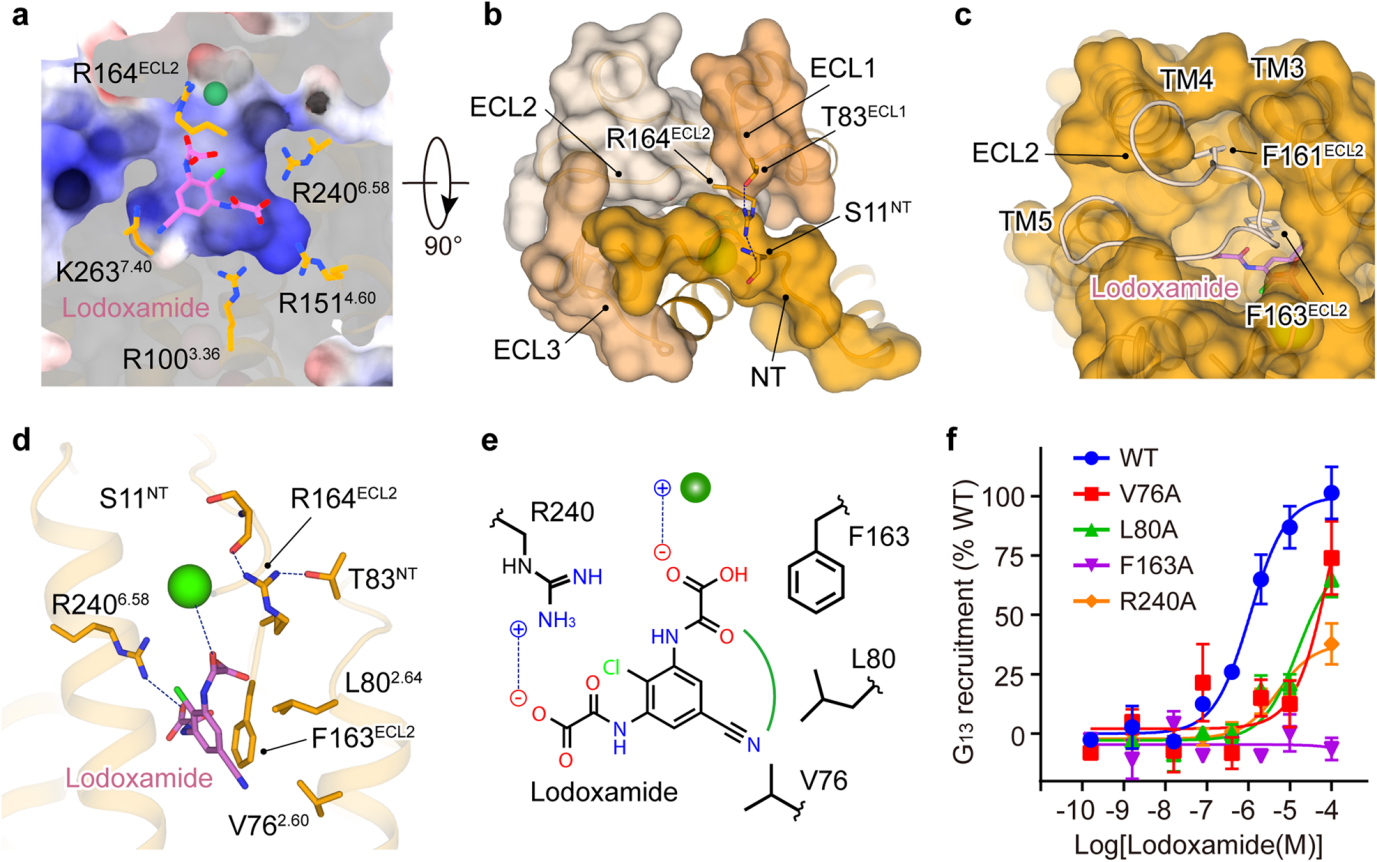
Lodoxamide recognition by GPR35. **a** Cross-section of the lodoxamide-binding pocket in GPR35. The pocket is colored by electrostatic surface potential, with the positive potential colored in blue. **b**, **c** The extracellular view of GPR35. The N-terminus (NT), all ECLs (ECL1, ECL2, and ECL3), which cover the ligand-binding pocket, are shown in a surface presentation (**b**). The polar interactions between these extracellular receptor components are indicated by blue dashed lines. ECL2 stretches into the ligand-binding pocket and stuffs the space embraced by lodoxamide and the extracellular portion of TM3, TM4, and TM5 (**c**). **d** Detailed interactions that contribute to lodoxamide binding in GPR35. The polar interactions are depicted by blue dashed lines. **e** 2D presentation of the interactions between lodoxamide and receptor. **f** Effects of pocket residue mutations on lodoxamide-induced G_13_ recruitment by GPR35.

Globally, lodoxamide occupies a conserved orthosteric binding pocket of class A GPCRs (Fig. 3a). The entire ligand-binding pocket of GPR35 is capped by the extracellular components, including the N-terminal loop and all three ECLs (Fig. 3b). Specifically, the N-terminal loop covers the center of the ligand-binding pocket. It is partially overlapped with the α-helical N-terminus of two lipid GPCRs, sphingosine-1-phosphate receptor subtype 1 (S1P1, PDB: 3V2Y) and lysophospholipid receptor 1 (LPA1, PDB: 4Z34), and highly resembles the N-terminal loop of C-X-C chemokine receptor type 4 (CXCR4, PDB: 3ODU) (Supplementary Fig. S5), thus highlighting the conformation diversity of the GPCR N-terminus. ECL2 of GPR35 stretches into the ligand-binding pocket and stuffs the space embraced by lodoxamide and the extracellular portion of TM3, TM4, and TM5. The side chains of two aromatic residues, F161^ECL2^ and F163^ECL2^, vertically point downwards and constitute the major interface of ECL2 with the ligand and receptor TM region. F161^ECL2^ stabilizes the extracellular segments of TM3 and TM4, while F163^ECL2^ forms a face-to-edge interaction with the phenylene group of the lodoxamide and substantially contributes to the ligand activity (Fig. 3c, f; Supplementary Table S3). The N-terminus and ECLs of GPR35 are linked by two intramolecular H-bonds (S11^NT^–R164^ECL2^ and T83^ECL1^–R164^ECL2^) and constitute a compact cap of the ligand-binding pocket (Fig. 3b, d).

Of particular note, GPR35 is featured by its strong, positively charged ligand-binding pocket, defined by several basic residues, including R100^3.36^, R151^4.60^, R164^ECL2^, R240^6.58^, and K263^7.40^ (Fig. 3a). Among these basic residues, only R240^6.58^ directly contacts lodoxamide by constituting a salt bridge with the 2-oxoacetic acid of the ligand. This positively charged binding pocket of GPR35 is theoretically compatible with acidic agonists with diverse sizes and chemical structures. Indeed, GPR35 is also responsible for other anti-asthma and anti-allergic agents, characterized by their symmetry diacids like lodoxamide, such as nedocromil sodium, bufrolin, and cromolyn disodium^21^. Positively charged residues, including R100^3.36^, R151^4.60^, R164^ECL2^, and R240^6.58^, are reported to be involved in the regulation of GPR35 activity by several agonists^21,41^. The non-specificity of the complementary electrostatic interaction between the ligand and the receptor may help explain the somewhat promiscuous ligand-binding mode of GPR35. Besides polar interactions, V76^2.60^, and L80^2.64^, together with F163^ECL2^, form hydrophobic interactions and contribute to lodoxamide-induced GPR35 activation (Fig. 3d–f; Supplementary Table S3).

### Activation mechanism of GPR35

**Fig. 4:**
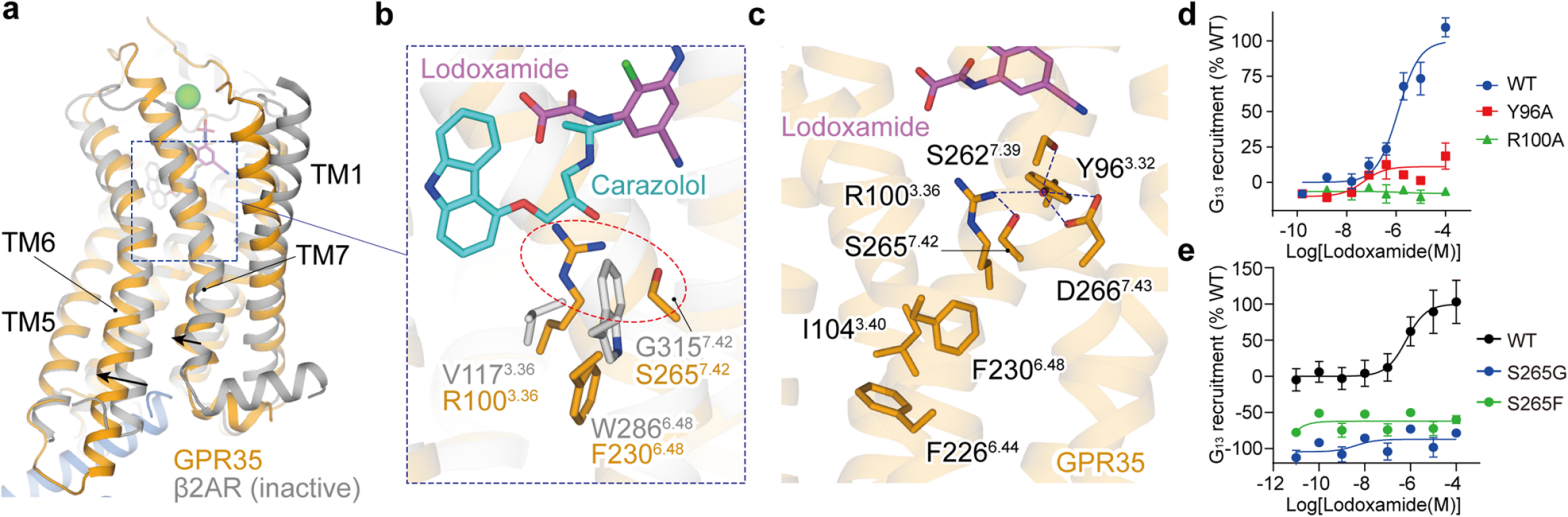
Activation mechanism of GPR35 by lodoxamide. **a** Structural superposition of GPR35 with the inactive β_2_AR. The movement directions of TM6 and TM7 of GPR35 (orange) relative to the inactive β_2_AR (grey, PDB: 2RH1) are indicated by black arrows. **b** The potential steric hindrance, caused by R100^3.36^ and S265^7.42^, pushes F230^6.48^ moving downward. The potential steric hindrance is highlighted in a red dashed circle. **c** The polar interaction network at the bottom of the ligand-binding pocket of GPR35 and the gain-of-inter-helical hydrophobic contacts between F230^6.48^ and two conserved PIF residues, I104^3.40^ and F226^6.44^. **d**, **e** Effects of mutation of residues in the polar interaction network on lodoxamide-induced G_13_ recruitment by GPR35.

Structural superposition of the lodoxamide–GPR35–G_13_ complex with a model class A GPCR, β_2_-adrenergic receptor (β_2_AR), in the inactive state (PDB: 2RH1) provides clues for understanding the basis for GPR35 activation. Compared with inactive β_2_AR, the cytoplasmic end of GPR35 TM6 undergoes a pronounced outward displacement, the hallmark of GPCR activation, and an inward movement of TM7 toward TM3^42^. This structural observation supports the contention that GPR35 is indeed in its active conformation (Fig. 4a).

The structure of lodoxamide-bound GPR35 offers a template to deduce the receptor activation mechanism. At the bottom of the ligand-binding pocket, the side chain of R100^3.36^ upright stretches and shallows the ligand-binding pocket of GPR35, thus hampering the probable insertion of the ligand and its further engagement with F230^6.48^, a toggle switch residue. Structural comparison of GPR35 with inactive β_2_AR reveals a potential steric hindrance of the side chain of R100^3.36^ and S265^7.42^ on F230^6.48^, which pushes the side chain of F230^6.48^ to move downward (Fig. 4b). Mutating R100^3.36^ to alanine abolished the lodoxamide-induced receptor activation (Fig. 4d; Supplementary Table S3). The conformational change of the F230^6.48^ side chain further leads to the gain-of-inter-helical hydrophobic contacts between F230^6.48^ and two conserved residues in the PIF micro-switch, I104^3.40^, and F226^6.44^ (Fig. 4c), thus initiating propagation of agonism signal and opening the cytoplasmic cavity to engage the extreme C-terminus of α5 helix of Gα_13_ subunit.

The structural inspection also reveals an extensive polar interaction network at the bottom of the ligand-binding pocket. Specifically, R100^3.36^ forms intramolecular polar interactions with Y96^3.32^ and S265^7.42^, where Y96^3.32^ also makes additional polar contacts with S262^7.39^ and D266^7.43^, thus forming a polar interaction network to fasten TM3 and TM7. This network structurally links the ligand-binding pocket to the cytoplasmic part of helices for G protein coupling and raises its probable role in downward transmitting ligand agonism signals (Fig. 4c). This speculation is corroborated by the abolished lodoxamide activity for the alanine mutants of R100^3.36^ and Y96^3.32^ (Fig. 4d) and is also supported by the previous report on the importance of R100^3.36^ and Y96^3.32^ in kynurenic acid- and zaprinast-induced GPR35 activation^43^. These findings support the critical role of these two residues in lodoxamide activity and also indicate the similarity of GPR35 activation mode by diverse agonists. Interestingly, substituting S265^7.42^ with glycine and phenylalanine almost completely abolished the lodoxamide activity, which may be attributed to the disturbance of the TM3 α-helical conformation by glycine and the dissociation of TM3 and TM7 by the bulkier phenylalanine (Fig. 4e).

The structure of the lodoxamide–GPR35–G_13_ complex also provides a template for understanding the rationale of disease-associated mutations. Several single nucleotide polymorphisms located in *gpr35* have been identified and are closely associated with IBDs and other immune- and inflammation-related diseases, of which the hyperactive T108^3.44^M mutation serves as the most frequent variant^44^. Structural inspection reveals that T108^3.44^ sits far from the ligand-binding pocket. Its methionine substitute may constitute a more powerful hydrophobic interaction with residues in TM4 (L140^4.49^) and TM5 (P183^5.50^ and V186^5.53^), thus providing additional stability relative to threonine in the wild-type receptor (Supplementary Fig. S6a). This structural observation supports previous findings that although T108^3.44^M showed a negligible effect on the potency of GPR35 agonists^21^, it increased baseline Ca^2+^ levels^28^. In addition, V76^2.60^M stands out as another disease-associated mutation. It is reasonable considering the ligand-binding pocket location of V76^2.60^ and its importance to activities of GPR35 agonists, such as lodoxamide, bufrolin, zaprinast, and cromolyn^21^ (Fig. 3d–f). The decrease of lodoxamide potency on the V76^2.60^M mutant of GPR35 is probably attributed to the steric hindrance between the bulkier side chain of methionine relative to valine and the phenylene group of lodoxamide (Supplementary Fig. S6b).

### G_13_ protein-coupling of GPR35

**Fig. 5:**
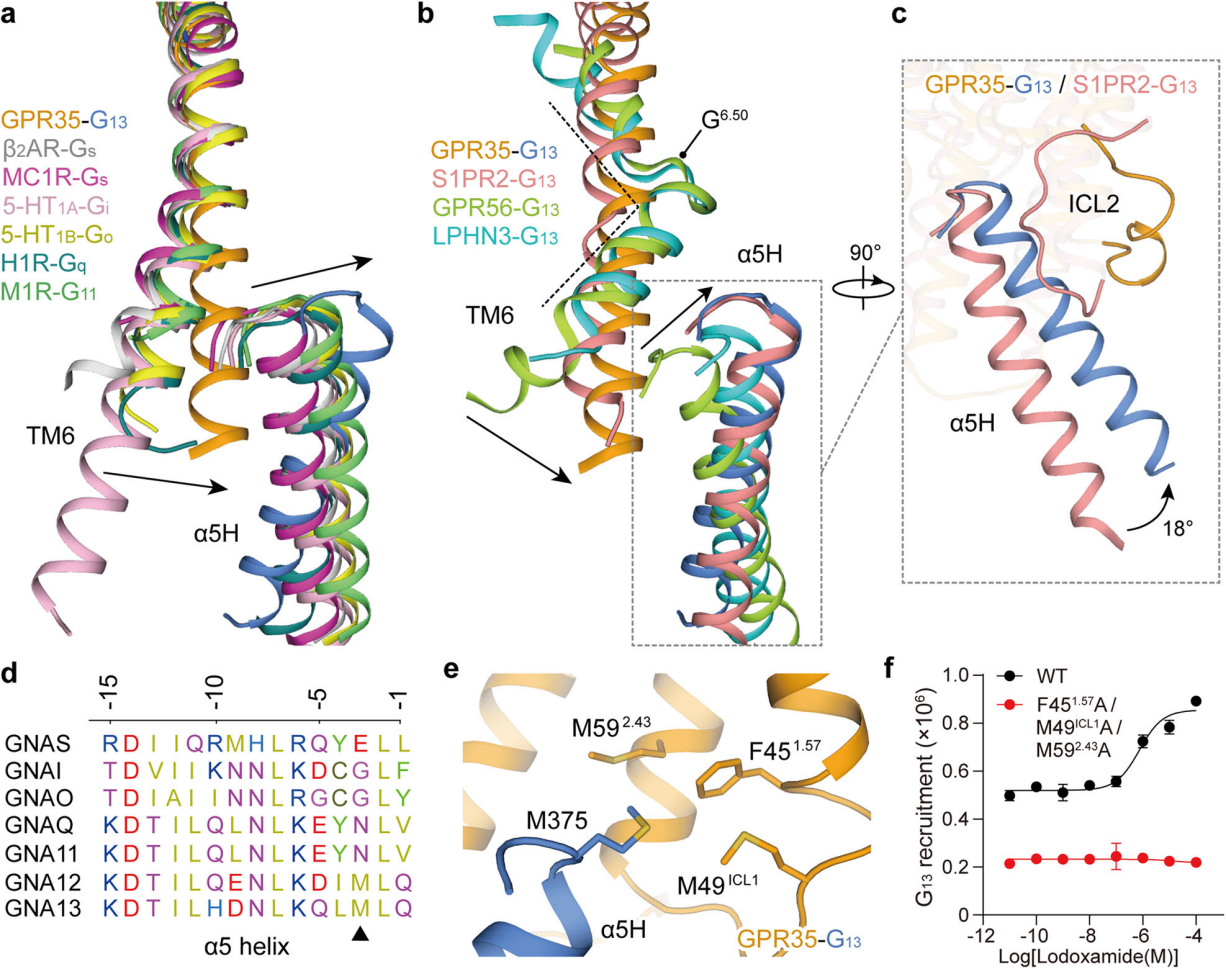
Mechanism of GPR35–G_13_ coupling. **a** Structural comparison of the GPR35–G_13_ complex with representative class A GPCRs coupled to G_s_, G_i/o_, and G_q/11_ proteins, including the β_2_AR–G_s_ (PDB: 3SN6), MC1R–G_s_ (PDB: 7F4F), 5-HT_1A_–G_i_ (PDB: 7E2Y), 5-HT_1B_–G_o_ (PDB: 6G79), H1R–G_q_ (PDB: 7DFL), and M1R–G_11_ complexes (PDB: 6OIJ). **b** Structural comparison of the GPR35–G_13_ complex with reported G_13_-coupled GPCRs, including class A GPCR S1PR2 (PDB: 7T6B) and two adhesion (class B2) GPCRs, GPR56 (PDB: 7SF8) and latrophilin 3 (LPHN3, PDB: 7SF7). The movement orientations of the cytoplasmic end of TM6 and the extreme C-terminal end of α5 helix of the Gα subunit in the GPR35–G_13_ complex compared with those of other listed GPCR–G protein complexes are indicated by black arrows. The black dashed line indicates the sharp kink in GPR56 and LPHN3. The hinge residue G^6.50^ was labeled. The GPCR–G protein complexes are colored as indicated. **c** The rotation of the α5 helix of the GPR35–G_13_ complex relative to that of the S1PR2–G_13_ complex. The rotation angle of 18° is indicated by a black arrow. The rotation probably arises from the extensive contacts between S1PR2 ICL2 and the α5 helix, which induces the noncanonical loop-like ICL2 to face inward towards the α5 and its resulting rotation. **d** Sequence alignment of C-terminal sequences of α5 helix of different G protein subtypes. The residues at the –3 position are labeled by a black triangle. **e** The methionine pocket in GPR35. M375 (–3) of G_13_ α5 helix forms hydrophobic interactions with residues F45^1.57^, M49^ICL1^, and M59^2.43^, which constitute a featured methionine pocket of GPR35. **f** Effects of mutations of residues in the methionine pocket on G_13_ recruitment by GPR35.

Compared with GPCRs coupled to the other three G protein subtypes, G_s_, G_i/o_, and G_q/11_, the smallest proportion of GPCRs engage with G_12/13_ protein. In contrast to GPCRs coupled to other G protein subtypes, GPR35 shows a similar G protein assembly mode and shares two major G protein-coupling interfaces, including a primary interface between the cytoplasmic cavity of receptor helices and α5 helix of the Gα_13_ subunit, and a hydrophobic interface between ICL2 of the receptor and αN and α5 of the Gα_13_ subunit (Supplementary Fig. S7a–d). However, the G_13_ protein shows several unique receptor-coupling features. The most striking difference is the relative position of the α5 helix of G_13_ protein and the concomitant shift of the cytoplasmic end of receptor TM6 (Fig. 5a). The extreme C-terminus of the Gα_13_ subunit in the GPR35–G_13_ complex shifts toward the core of the cytoplasmic cavity of the receptor (7.0–7.8 Å, 5.7–6.8 Å, and 5.3–5.5 Å for Gα_s_, Gα_i/o_, and Gα_q/11_ subunit, respectively, measured at the Cα atom of L (–2), α5 helix numbering starts with –1 from the terminal residue). On the receptor side, the inward displacement of the Gα_13_ C-terminus is associated with the smallest outward displacement of TM6 relative to GPCRs coupled to the other three G protein subtypes (8.5–9.7 Å, 7.6–8.2 Å, and 6.4–10.2 Å for G_s_-, G_i/o_-, and G_q/11_-coupled GPCRs, respectively, measured at the Cα of residue at 6.30) (Fig. 5a).

Recently, three cryo-EM structures of engineered G_13_-coupled GPCRs, including a class A GPCR sphingosine-1-phosphate receptor 2 (S1PR2, PDB: 7T6B) and two adhesion (class B2) GPCRs GPR56 (PDB: 7SF8) and latrophilin 3 (LPHN3, PDB: 7SF7), were reported^16,17^, which provide structural templates to characterize G_13_ recruitment features across different receptors. G_13_-coupled GPR35 and S1PR2 (PDB: 7T6B) show similar relative positions of the receptor TM6 (Fig. 5b). Noteworthily, the α5 helix of the GPR35–G_13_ complex undergoes a ~18° rotation upward from the pivot point of the extreme C-terminus of the α5 helices of Gα_13_ subunits relative to the S1PR2–G_13_ complex. The ICL2 of S1PR2 makes extensive contacts with the α5 helix, which induces the noncanonical loop-like ICL2 to face inward towards the α5 and its resulting rotation (Fig. 5c). More remarkable conformation differences were seen between G_13_-coupled GPR35 and two adhesion GPCRs. The α5 helix and cytoplasmic end of TM6 of the GPR35–G_13_ complex stay in a position approaching the receptor helix core relative to the G_13_-coupled GPR56 and LPHN3 complexes. The unique activation feature of GPR56 and LPHN3, the sharp kink of TM6 at the hinge residue G^6.50^, would be responsible for these conformational distinctions, highlighting the class-specific manner of GPCRs for G_13_ recruitment (Fig. 5b).

We further assess the contribution of the Gα_13_ C-terminus to GPR35 engagement by mutating residues at the receptor interface. C-terminal residues of Gα_13_, completely conserved or sharing similar physiochemical properties across four G protein subtypes, including L376 (–2), L374 (–4), L371 (–7), and N370 (–8), are essential for GPR35 coupling by forming hydrophobic and van der Waals interactions (Supplementary Fig. S7e and Table S4). In addition, the side chain of the conserved D364 (–14) constitutes a stabilizing salt bridge with R201^5.68^ of GPR35. The corresponding salt bridges also exist in G_i_-coupled 5-HT_1A_ (PDB: 7E2Y), G_o_-coupled 5-HT_1B_ (PDB: 6G79), and G_11_–M1R (PDB: 6OIJ) complexes; alternatively, the conserved D364 (–14) forms similar polar contact with cognate Q^5.68^ (β_2_AR–G_s_, PDB: 3SN6 and H1R–G_q_, PDB: 7DFL) or H^5.68^ (MC1R–G_s_, PDB: 7F4F) (Supplementary Fig. S7f). Despite that, the side chain of R201^5.68^ in GPR35 exerts a significantly different conformational change, showing a rotation towards TM6 relative to other receptors. This large amplitude rotation reorients the side chain of R201^5.68^, turning it towards the α5 helix of Gα_13_, thus creating a possible crash with the C-terminal residue (–1) and the entire α5 helix of other G protein subtypes. The caused steric hindrance further leads to the tilt of the α5 helix and the shift of the extreme C-terminus of the Gα_13_ α5 helix in contrast to other G protein subtypes (Supplementary Fig. S7f). The structural observation is supported by the dramatically decreased lodoxamide activity on the R201^5.68^A mutant of GPR35 (Supplementary Fig. S7g).

The sequence alignment of different G protein subtypes reveals a poorly conserved residue M375 (–3) at the C-terminus of G_12/13_ protein (Fig. 5d). The relative inward displacement of the Gα_13_ C-terminus results in the vicinity of M375 (–3) to the cytoplasmic parts of receptor TM1, ICL1, and TM2. The side chain of M375 (–3) is thus engaged in a hydrophobic sub-pocket enriched with methionine (F45^1.57^, M49^ICL1^, and M59^2.43^), designated as the ‘methionine pocket’ (Fig. 5e). Substitution of M375 (–3) with alanine or charged residues arginine and lysine dramatically decreased the activity of lodoxamide (Supplementary Fig. S7e). The abolished ligand potency on triple alanine mutations of F45^1.57^, M49^12.48^, and M59^2.43^ further supports the critical role of this ‘methionine pocket’ in GPR35–G_13_ coupling (Fig. 5f). Together, combined with G protein recruitment analysis, our structure reveals several unique structural features on G_13_ coupling and a featured ‘methionine pocket’, thus providing the molecular basis for understanding the mechanism of GPCR–G_13_ coupling.

## Discussion

Mg^2+^ and Ca^2+^ regulate multiple physiological functions, and magnesium and calcium homeostasis are fundamental to the existence of life. The imbalance of magnesium (hypomagnesemia and hypermagnesemia) and calcium (hypocalcemia and hypercalcemia) results in severe human diseases. In the cryo-EM structure of the lodoxamide–GPR35–G_13_ complex, we observed a strong spheroid EM density approaching the extracellular surface of the receptor, sitting in a distinctive site compared with other reported cation sites in class A GPCRs. The density is further identified to respond to divalent cations Mg^2+^ or Ca^2+^ by testing their allosteric agonism effects on GPR35. The effective concentrations of cations used under our experimental condition are 1–40-fold over the average physiological concentrations. However, regardless of the sensitivity discrepancy of different in vitro assays, this higher cation concentration in the local intestine is accessible since the GPR35-enriched intestine is responsible for cation absorption. From an evolutionary perspective, the requirement for higher cation concentration guarantees the precise regulation of GPR35 in the local intestine. In addition, it was reported that IBDs could induce functional defection and/or decreased expression of epithelial Na^+^-K^+^-ATPase, Na^+^/H^+^ exchangers, epithelial Na^+^ channels, and K^+^ channels, which disrupt the hydroelectrolytic homeostasis by lowering Na^+^ absorption and/or increasing K^+^ secretion, eventually leading to diarrhea, the most common symptom of IBD^29,30^. Under our experimental conditions, GPR35 does not appear to be affected by either Na^+^ or K^+^. These findings give a hint, which still remains to be identified, that divalent cations Mg^2+^ and Ca^2+^ may also be involved in IBD pathogenesis in a different manner by regulating GPR35 but not the Na^+^ and K^+^ balance.

In addition, our structure demonstrates a highly positively-charged ligand-binding pocket of GPR35 and reveals the mechanism of receptor recognition and activation by lodoxamide. The electrostatic complementary binding mode may explain the binding promiscuity for GPR35 by diverse acidic metabolites. Meanwhile, the relatively weaker activity of these metabolites for GPR35 at the micromolar scale is evolutionarily economic, as the metabolites are primarily absorbed in the GPR35-highly-expressed intestine. Structural comparison of the GPR35–G_13_ complex with GPCR coupled to other three G protein subtypes (G_s_, G_i_, and G_q/11_) reveals two striking structural features: the shift of the C-terminus of Gα_13_ α5 helix towards the core of the cytoplasmic cavity of receptors and the concomitant inward movement of the cytoplasmic end of receptor TM6, which may be stemmed from the potential steric hindrance between R201^5.68^ and the terminal residue (–1) and the entire α5 helix. We further identify a featured ‘methionine pocket’ in GPR35, which accommodates the G_13_-specific residue M375 (–3) at the C-terminus of G_12/13_ protein.

Besides G_12/13_ protein, GPR35 was thought to interact with the inhibitory G_i/o_ protein. The initial evidence came from chimeric G proteins by displacing the C-terminal amino acids of Gα_q_ or promiscuous Gα_15/16_ by those of G_i/o_, which redirects the cAMP accumulation signal of G_i/o_ to the calcium mobilization signal^19,45^. The direct inhibition effects of forskolin-induced cAMP accumulation in response to GPR35 agonists were also observed^46^. These reports support a potential G_i/o_-coupling activity of GPR35. However, no coherent attempt and systematic functional analysis have yet been made to assess if these agonists promote GPR35 recruiting G_12/13_ or G_i/o_ differentially. Compared with representative GPCRs coupled to G_s_, G_i/o_, and G_q/11_, a smaller outward movement of the cytoplasmic end of TM6 of GPR35 when coupled to G_13_ may produce a smaller cavity on the cytoplasmic side of the receptor heptahelical domain, theoretically disfavoring the accommodation of Gα_i/o_, which own the bulkiest C-terminal residues across Gα subunits (F354/Y354 for Gα_i/o_, Fig. 5d and Supplementary Fig. S7f). Thus, a different G_i/o_-coupling mechanism for GPR35 compared with G_13_ protein is anticipated, which could be unveiled by the GPR35–G_i/o_ complex structure. Interestingly, β-arrestin 2 recruitment assay was reported to show a high signal-to-background ratio and had been widely employed for deorphanization or ligand activity evaluation for GPR35^21,41,43^. However, in order to understand GPR35 signal transduction, it is still necessary to accurately identify ligand ‘bias’ and the mechanism of β-arrestin 2 recruitment by GPR35. In summary, the structure of the lodoxamide–GPR35–G_13_ complex offers insights into a novel binding site and a different regulatory model of divalent cations. The complementary electrostatic ligand recognition mode provides a clue for designing drugs against IBDs. Our structure also provides a framework for understanding the rationale of specific G_13_ coupling by GPCRs.

## Materials and methods

### Expression and purification of the GPR35–G_13_ protein complex

The full-length human GPR35 (residues 1–309) was applied for cryo-EM studies. GPR35 cDNA was cloned into a modified pFastBac vector (Invitrogen) containing the N-terminal thermal-stabilized BRIL^47^ to enhance receptor expression and the N-terminal Flag/His tag. The tobacco etch virus (TEV) protease recognition site was inserted in front of the receptor sequence. The N-terminus of human Gα_13_ was replaced by Gα_i1_ (residues 1–18) to facilitate Gα_13_ expression^48^ and the scFv16 binding^31^. The NanoBiT tethering strategy was applied to obtain a stable GPR35–G_13_ complex^32^. All constructs were prepared using homologous recombination (CloneExpress One Step Cloning Kit, Vazyme). Receptor-LgBiT, Gα_13,_ rat H6-Gβ-HiBiT, bovine Gγ, scFv16 were co-expressed in *Spodoptera frugiperda* (*sf9*) insect cells (Invitrogen).

Cell pellets of the co-expression culture were thawed and lysed in 20 mM HEPES, pH 7.4, 100 mM NaCl, 10% glycerol, 5 mM MgCl_2_, and 10 mM CaCl_2_ supplemented with EDTA-Free Protease Inhibitor Cocktail (TargetMol). The GPR35–G_13_ complex was assembled at room temperature for 1 h by the addition of 20 μM lodoxamide and 25 mU/mL apyrase. The lysate was then solubilized in 0.5% LMNG, 0.1% CHS, and the soluble fraction was then incubated with Talon affinity resin for 2 h. After extensive washing, TEV protease was added and incubated for 1 h at room temperature. The flow-through was collected, concentrated, and injected onto a Superdex 200 10/300 column equilibrated in the buffer containing 20 mM HEPES, pH 7.4, 100 mM NaCl, 0.00075% LMNG, 0.00025% GDN, 0.0002% CHS, and 20 μM lodoxamide. The monomeric complex peak was collected and concentrated to ~6 mg/mL for cryo-EM studies.

### Cryo-EM grid preparation and image collection

For the preparation of cryo-EM grids, 2.5 µL of the purified GPR35–Gα_13_ protein complex was applied individually onto the glow-discharged holey carbon grids (Quantifoil, Au300 R1.2/1.3) in a Vitrobot chamber (FEI Vitrobot Mark IV). The Vitrobot chamber was set to 100% humidity at 4 °C. Extra samples were blotted for 2 s and were vitrified by plunging into liquid ethane. Grids were stored in liquid nitrogen for condition screening and data collection usage.

Automatic data collection of the GPR35–Gα_13_ protein complex was performed on an FEI Titan Krios G4 operated at 300 kV. The microscope was operated with a nominal magnification of 81,000× in super-resolution mode, corresponding to a pixel size of 1.04 Å for the micrographs. A total of 17,605 movies for the dataset of the GPR35–Gα_13_–scFv16 complex were collected by a Gatan K3 Summit direct electron detector with a Gatan energy filter (operated with a slit width of 20 eV) (GIF) using the EPU software. The images were recorded at a dose rate of about 15.0 e/Å^2^/s with a defocus ranging from –0.5 to –2.0 μm. The total exposure time was 3.02 s resulting in a total of 36 frames per micrograph.

### Cryo-EM data processing

Cryo-EM image stacks were aligned using Relion^49^. Contrast Transfer Function (CTF) parameters for each micrograph were estimated by CTFFIND4.1^50^. The following data processing was performed using RELION-4.0-beta. For the lodoxamide–GPR35–G_13_–scFv16 complex, automated particle selection yielded 13,462,594 particle projections. The projections were subjected to reference-free 2D classification to discard poorly defined particles, producing 665,480 particle projections for three-dimensional classification with a pixel size of 1.04 Å. Further 3D classification focusing the alignment on the receptor produced one good subset accounting for 510,197 particles, which were subsequently subjected to 3D refinement, CTF refinement, and Bayesian polishing. The final refinement generated a map with an indicated global resolution of 3.2 Å and was subsequently post-processed by DeepEMhancer^51^.

### Model building and refinement

All PDB coordinates using alphafold2^52^ served as a starting model for building the atomic model. Models were docked into the EM density map using UCSF Chimera^53^, followed by iterative manual adjustment and rebuilding in COOT^54^. Real space and reciprocal space refinements were performed using Phenix programs. The model statistics were validated using Rosetta. The final refinement statistics were validated using the module “comprehensive validation (cryo-EM)” in Phenix^55^. The final refinement statistics are provided in Supplementary Table S1.

### NanoBiT assay for G_13_ recruitment

For the G_13_ recruitment assay performed with insect cells, all the constructs were similar to the expression constructs except the Gβ subunit with a SmBiT at its N-terminus. Receptor-LgBiT, Gα_13_, rat H6-Gβ-SmBiT, bovine Gγ, and GST-Ric-8A were co-expressed in *sf9* insect cells for 48 h. The following procedures were performed similarly to the β-arrestin recruitment assay, except that the 10 μL 50 mM coelenterazine was added for detection instead of the NanoLuc substrate (furimazine).

For cation detection, insect cells were broken, and cell membranes were collected for the assay. Membranes were diluted 50 times using dilution buffer (20 mM HEPES, 100 mM NaCl, pH 7.5) before being seeded in a 384-well plate. The 10 μL ligands and 10 μL cations were added to the cells, respectively. The GPR35 receptor treated with 1 mM EDTA throughout the procedure and without the addition of any cations is defined as control. The membranes were incubated at 37 °C for 30 min before 10 μL 50 mM coelenterazine was added for detection.

### Detection of surface expression of GPR35 mutants

The surface expression detection was performed in AD293 cells. Cells were maintained at 37 °C in a 5% CO_2_ incubator with 150,000 cells per well in a 12-well plate. Cells were grown overnight and then transfected with 1.0 μg GPR35 construct by FuGENE^®^ HD transfection reagent in each well for 24 h. After 24 h of transfection, cells were washed once with PBS and then detached with 0.2% (w/v) EDTA in PBS. Cells were blocked with PBS containing 5% (w/v) BSA for 15 min at room temperature before incubating with primary anti-Flag antibody (diluted with PBS containing 5% BSA at a ratio of 1:300, Sigma) for 1 h at room temperature. Cells were then washed three times with PBS containing 1% (w/v) BSA and then incubated with anti-mouse Alexa-488-conjugated secondary antibody (diluted at a ratio of 1:1000, Invitrogen) at 4 °C in the dark for 1 h. After another three times of washing, cells were collected, and fluorescence intensity was quantified in a BD Accuri C6 flow cytometer system (BD Biosciences) through a BD Accuri C6 software 1.0.264.21 at excitation of 488 nm and emission of 519 nm. Approximately 10,000 cellular events per sample were collected, and data were normalized to the wild-type GPR35. Experiments were performed at least three times, and data were presented as means ± SEM.

### Quantification and statistical analysis

All functional study data were analyzed using Prism 8 (GraphPad) and presented as means ± SEM from at least three independent experiments. Concentration-response curves were evaluated with a three-parameter logistic equation. pEC_50_ is calculated with the Sigmoid three-parameter equation. The significance was determined with two-side, one-way ANOVA with Tukey’s test, and *P < 0.01, **P < 0.001, and ***P < 0.0001 vs the wild type was considered statistically significant.

## Supplementary information

Supplementary Information

## Data Availability

The atomic coordinates and the electron microscopy map of the lodoxamide–GPR35–G_13_–scFv16 complex have been deposited in the Protein Data Bank (PDB) and Electron Microscopy Data Bank (EMDB) under accession codes 8H8J and EMD-34549, respectively.

## References

[1] Zarzycka B, Zaidi SA, Roth BL, Katritch V (2019). Harnessing ion-binding sites for GPCR pharmacology. Pharm. Rev..

[2] Liu W (2012). Structural basis for allosteric regulation of GPCRs by sodium ions. Science.

[3] Miller-Gallacher JL (2014). The 2.1 A resolution structure of cyanopindolol-bound beta1-adrenoceptor identifies an intramembrane Na+ ion that stabilises the ligand-free receptor. PLoS One.

[4] Zhang C (2012). High-resolution crystal structure of human protease-activated receptor 1. Nature.

[5] Fenalti G (2014). Molecular control of delta-opioid receptor signalling. Nature.

[6] Ma S (2021). Structural mechanism of calcium-mediated hormone recognition and Gbeta interaction by the human melanocortin-1 receptor. Cell Res.

[7] Yu J (2020). Determination of the melanocortin-4 receptor structure identifies Ca(2+) as a cofactor for ligand binding. Science.

[8] Hu X, Provasi D, Ramsey S, Filizola M (2020). Mechanism of mu-opioid receptor-magnesium interaction and positive allosteric modulation. Biophys. J..

[9] Chan HCS (2020). Enhancing the signaling of GPCRs via orthosteric ions. ACS Cent. Sci..

[10] Waltenspuhl Y, Schoppe J, Ehrenmann J, Kummer L, Pluckthun A (2020). Crystal structure of the human oxytocin receptor. Sci. Adv..

[11] Siehler S (2007). G12/13-dependent signaling of G-protein-coupled receptors: disease context and impact on drug discovery. Expert Opin. Drug Disco..

[12] Worzfeld T, Wettschureck N, Offermanns S (2008). G(12)/G(13)-mediated signalling in mammalian physiology and disease. Trends Pharm. Sci..

[13] Syrovatkina V, Huang XY (2019). Signaling mechanisms and physiological functions of G-protein Galpha13 in blood vessel formation, bone homeostasis, and cancer. Protein Sci..

[14] Yang YM, Kuen DS, Chung Y, Kurose H, Kim SG (2020). Galpha12/13 signaling in metabolic diseases. Exp. Mol. Med..

[15] Rasheed SAK (2022). The emerging roles of Galpha12/13 proteins on the hallmarks of cancer in solid tumors. Oncogene.

[16] Chen H (2022). Structure of S1PR2-heterotrimeric G13 signaling complex. Sci. Adv..

[17] Barros-Alvarez X (2022). The tethered peptide activation mechanism of adhesion GPCRs. Nature.

[18] Mackenzie AE (2019). Receptor selectivity between the G proteins Galpha12 and Galpha13 is defined by a single leucine-to-isoleucine variation. FASEB J..

[19] Wang J (2006). Kynurenic acid as a ligand for orphan G protein-coupled receptor GPR35. J. Biol. Chem..

[20] De Giovanni M (2022). GPR35 promotes neutrophil recruitment in response to serotonin metabolite 5-HIAA. Cell.

[21] MacKenzie AE (2014). The antiallergic mast cell stabilizers lodoxamide and bufrolin as the first high and equipotent agonists of human and rat GPR35. Mol. Pharm..

[22] Uhlen M (2015). Proteomics. Tissue-based map of the human proteome. Science.

[23] Agudelo LZ (2018). Kynurenic Acid and Gpr35 Regulate Adipose Tissue Energy Homeostasis and Inflammation. Cell Metab..

[24] Imielinski, M. et al. Common variants at five new loci associated with early-onset inflammatory bowel disease. *Nat. Genet.* **41**, 1335–1340 (2009). 10.1038/ng.489 PMC3267927 19915574

[25] Ellinghaus D (2013). Genome-wide association analysis in primary sclerosing cholangitis and ulcerative colitis identifies risk loci at GPR35 and TCF4. Hepatology.

[26] Kaya B, Melhem H, Niess JH (2021). GPR35 in intestinal diseases: from risk gene to function. Front. Immunol..

[27] Liu JZ (2015). Association analyses identify 38 susceptibility loci for inflammatory bowel disease and highlight shared genetic risk across populations. Nat. Genet..

[28] Schneditz G (2019). GPR35 promotes glycolysis, proliferation, and oncogenic signaling by engaging with the sodium potassium pump. Sci. Signal..

[29] Magalhaes D, Cabral JM, Soares-da-Silva P, Magro F (2016). Role of epithelial ion transports in inflammatory bowel disease. Am. J. Physiol. Gastrointest. Liver Physiol..

[30] Anbazhagan AN, Priyamvada S, Alrefai WA, Dudeja PK (2018). Pathophysiology of IBD associated diarrhea. Tissue Barriers.

[31] Koehl A (2018). Structure of the micro-opioid receptor-Gi protein complex. Nature.

[32] Duan J (2020). Cryo-EM structure of an activated VIP1 receptor-G protein complex revealed by a NanoBiT tethering strategy. Nat. Commun..

[33] Cao C (2018). Structural basis for signal recognition and transduction by platelet-activating-factor receptor. Nat. Struct. Mol. Biol..

[34] Khrustalev VV, Barkovsky EV, Khrustaleva TA (2016). Magnesium and manganese binding sites on proteins have the same predominant motif of secondary structure. J. Theor. Biol..

[35] Thimm D, Funke M, Meyer A, Muller CE (2013). 6-Bromo-8-(4-[(3)H]methoxybenzamido)-4-oxo-4H-chromene-2-carboxylic Acid: a powerful tool for studying orphan G protein-coupled receptor GPR35. J. Med. Chem..

[36] Romani AM (2011). Cellular magnesium homeostasis. Arch. Biochem. Biophys..

[37] Atchison DK, Beierwaltes WH (2013). The influence of extracellular and intracellular calcium on the secretion of renin. Pflug. Arch..

[38] Meyerowitz JG (2022). The oxytocin signaling complex reveals a molecular switch for cation dependence. Nat. Struct. Mol. Biol..

[39] Ye L (2018). Mechanistic insights into allosteric regulation of the A2A adenosine G protein-coupled receptor by physiological cations. Nat. Commun..

[40] Zheng H, Chruszcz M, Lasota P, Lebioda L, Minor W (2008). Data mining of metal ion environments present in protein structures. J. Inorg. Biochem..

[41] Zhao P (2014). Crucial positively charged residues for ligand activation of the GPR35 receptor. J. Biol. Chem..

[42] Zhou Q (2019). Common activation mechanism of class A GPCRs. Elife.

[43] Jenkins L (2011). Agonist activation of the G protein-coupled receptor GPR35 involves transmembrane domain III and is transduced via Galpha(1)(3) and beta-arrestin-2. Br. J. Pharm..

[44] Quon T, Lin LC, Ganguly A, Tobin AB, Milligan G (2020). Therapeutic opportunities and challenges in targeting the orphan G Protein-Coupled Receptor GPR35. ACS Pharm. Transl. Sci.

[45] Taniguchi Y, Tonai-Kachi H, Shinjo K (2006). Zaprinast, a well-known cyclic guanosine monophosphate-specific phosphodiesterase inhibitor, is an agonist for GPR35. FEBS Lett..

[46] Kaya B (2020). Lysophosphatidic acid-mediated GPR35 signaling in CX3CR1(+) macrophages regulates intestinal homeostasis. Cell Rep..

[47] Chun E (2012). Fusion partner toolchest for the stabilization and crystallization of G protein-coupled receptors. Structure.

[48] Kreutz B (2006). A new approach to producing functional G alpha subunits yields the activated and deactivated structures of G alpha(12/13) proteins. Biochemistry.

[49] Zivanov J, Nakane T, Scheres SHW (2020). Estimation of high-order aberrations and anisotropic magnification from cryo-EM data sets in RELION-3.1. IUCrJ.

[50] Rohou A, Grigorieff N (2015). CTFFIND4: Fast and accurate defocus estimation from electron micrographs. J. Struct. Biol..

[51] Sanchez-Garcia R (2021). DeepEMhancer: a deep learning solution for cryo-EM volume post-processing. Commun. Biol..

[52] Senior AW (2020). Improved protein structure prediction using potentials from deep learning. Nature.

[53] Goddard TD, Huang CC, Ferrin TE (2007). Visualizing density maps with UCSF Chimera. J. Struct. Biol..

[54] Emsley P, Cowtan K (2004). Coot: model-building tools for molecular graphics. Acta Crystallogr. D Biol. Crystallogr..

[55] Adams PD (2010). PHENIX: a comprehensive Python-based system for macromolecular structure solution. Acta Crystallogr. D Biol. Crystallogr..

