# Supplementary material for: Insights into divalent cation regulation and G13-coupling of orphan receptor GPR35
Source: Cell Discov. 2022 Dec 21;8:135. doi: 10.1038/s41421-022-00499-8 (PMC9772185; doi:10.1038/s41421-022-00499-8)
Supplement: Supplementary file 1 — Supplementary Information [file 41421_2022_499_MOESM1_ESM.pdf]

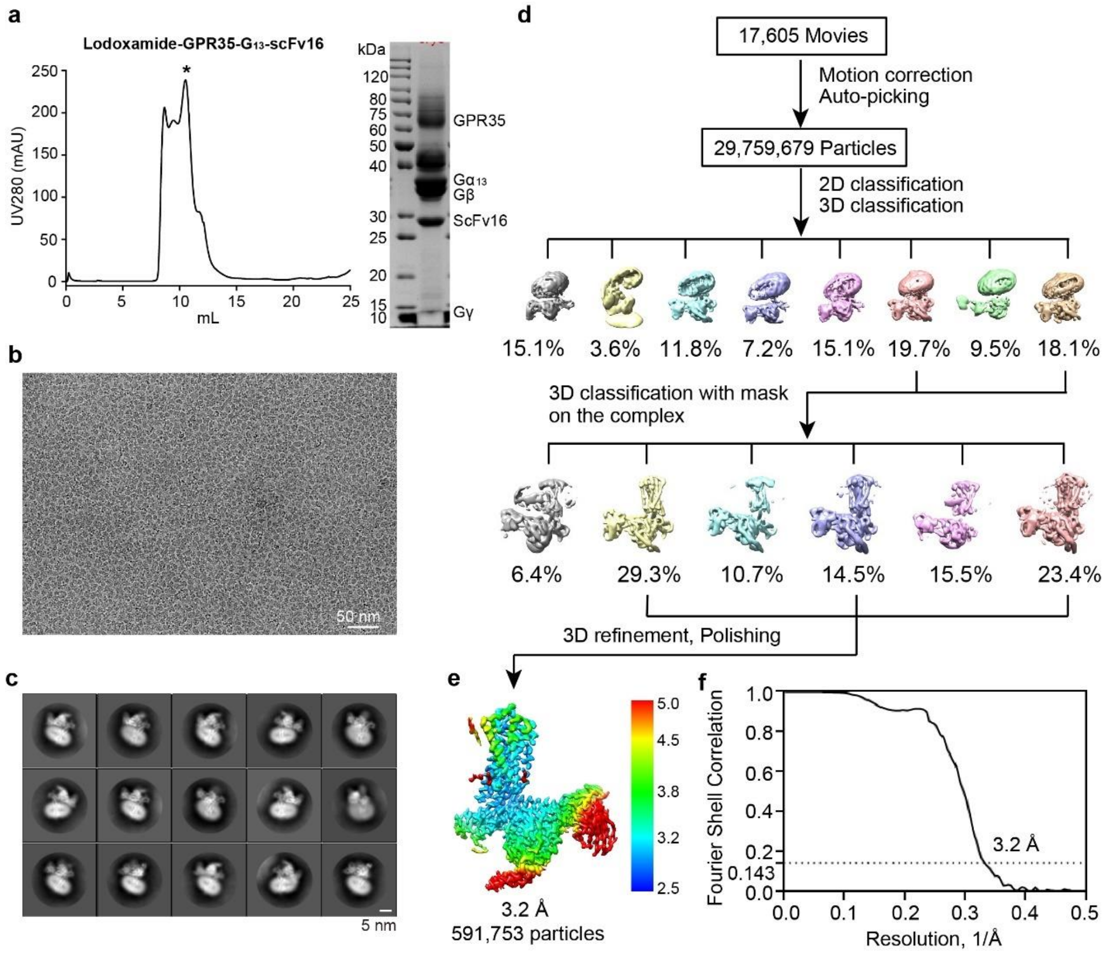

Fig. S1 The lodoxamide-GPR35-G_{13}-scFv16 complex purification and cryo-EM data processing.

a Representative elution profile of the lodoxamide-GPR35-G_{13}-scFv16 complex and SDS–PAGE of the size-exclusion chromatography peak. **b, c** Representative cryo-EM micrographs of the lodoxamide-GPR35-G_{13}-scFv16 complex (**b**, scale bar: 50 nm) and 2D class averages (**c**, scale bar: 5 nm). **d** Flow chart of cryo-EM data processing of the lodoxamide-GPR35-G_{13}-scFv16 complex. **e** 3D density map of the GPR35 complex colored according to local resolution (Å). **f** Gold-standard Fourier Shell Correlation (FSC) curve of the global non-uniform refinement of the complex.

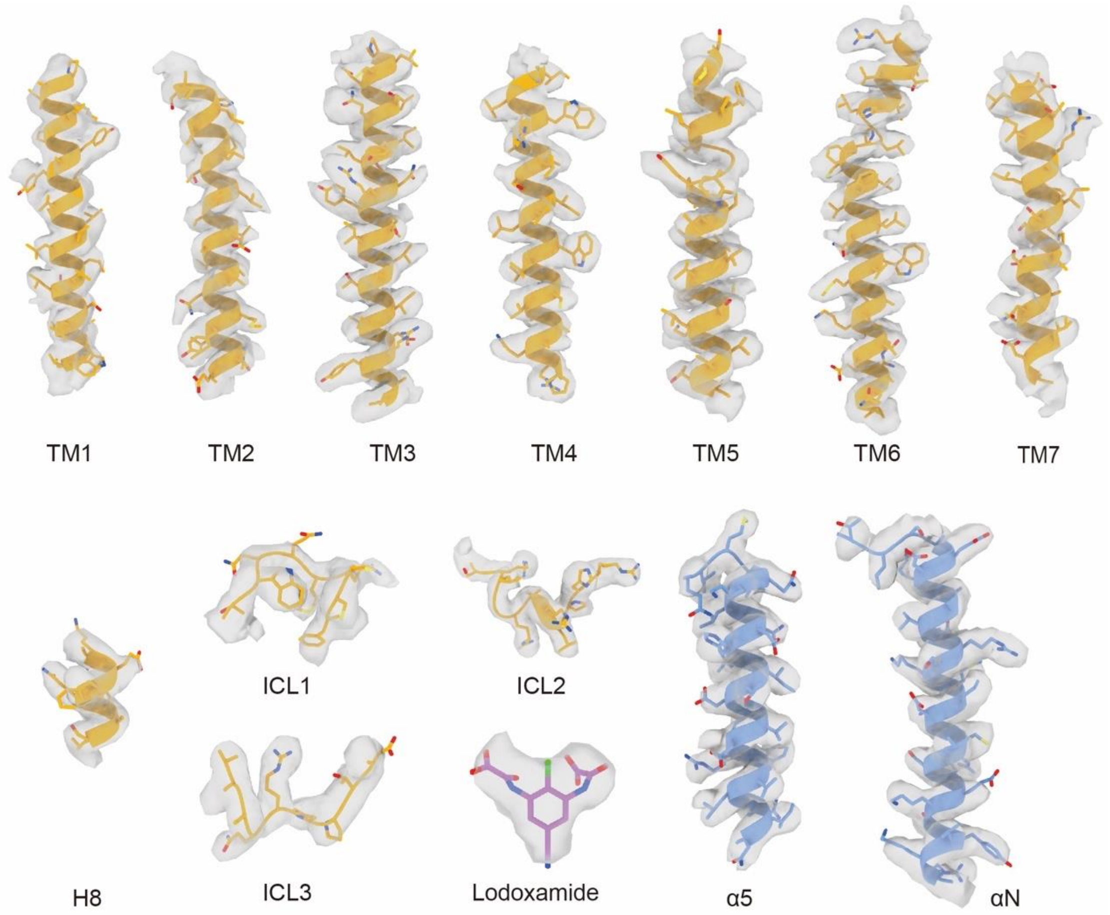

Fig. S2 Representative EM density and coordinate of the lodoxamide-GPR35-G$_{13}$-scFv16 complex.

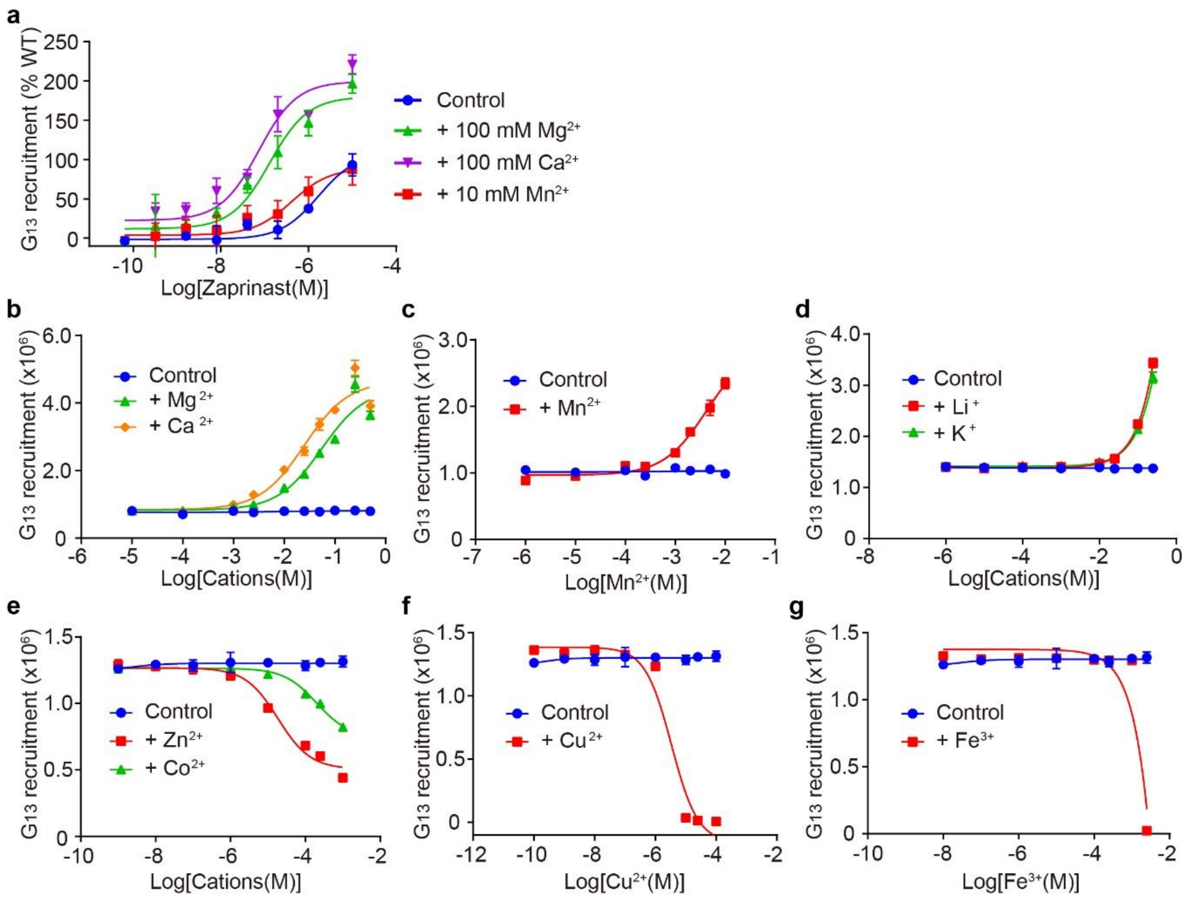

Fig. S3 Allosteric agonism effects of cations on zaprinast for GPR35 and their effects on the activity of apo GPR35.

a Allosteric agonism effects of divalent cations (Mg^{2+}, Ca^{2+}, and Mn^{2+}) on zaprinast for GPR35. **b-g** Effects of cations on apo GPR35 activity, including Mg^{2+}, Ca^{2+} (**b**), Mn^{2+} (**c**), Li^{+}, K^{+} (**d**), Zn^{2+}, Co^{2+} (**e**), Cu^{2+} (**f**), and Fe^{3+} (**g**). The GPR35 pretreated with EDTA and without the addition of any cations is defined as control.

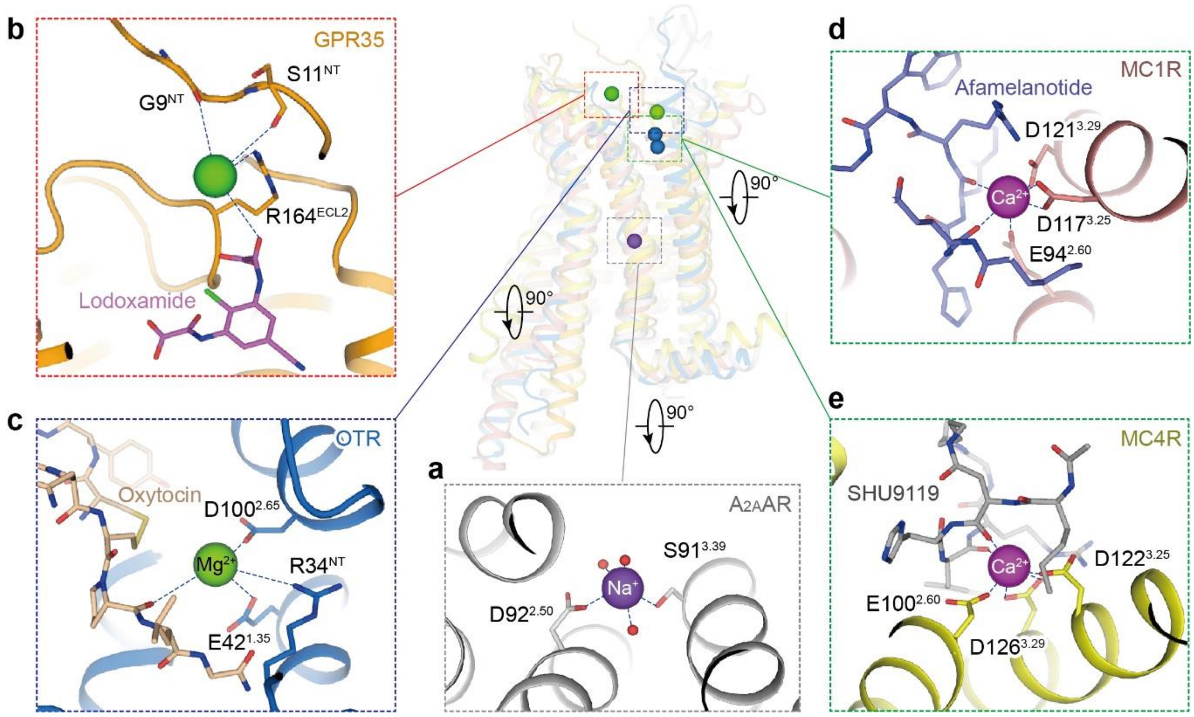

Fig. S4 Comparison of cation coordination sites in class A GPCRs.

a Na^+ coordination site buried in the TMD helices of $A_{2A}AR$ (PDB: 4EIY). The Na^+ site is highly conserved across class A GPCRs. **b** The divalent cation binding site in GPR35. **c** Mg^{2+} coordination site in oxytocin bound oxytocin receptor (OTR, PDB: 7RYC). **d** Ca^{2+} coordination site in afamelanotide bound melanocortin receptor 1 (MC1R, PDB: 7F4H). **e** Ca^{2+} coordination site in SHU9119 bound melanocortin receptor 4 (MC4R, PDB: 6W25). Polar interactions between cations and their coordinate residues are indicated by blue dashed lines.

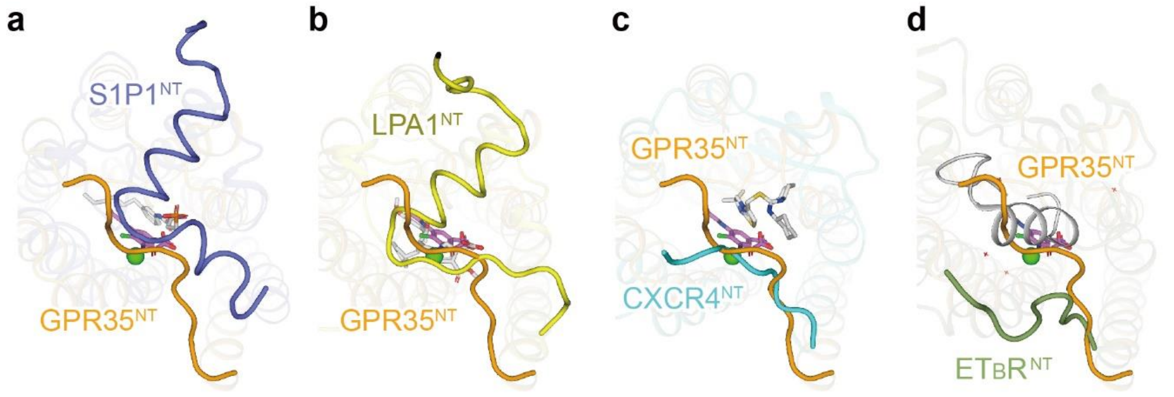

Fig. S5 Structural comparison of N-terminus in representative class A GPCRs.
Structural comparison of N-terminus (NT) of GPR35 with that of sphingosine-1-phosphate receptor subtype 1 (S1P1, PDB: 3V2Y, **a**), lysophospholipid receptor 1 (LPA1, PDB: 4Z34, **b**), C-X-C chemokine receptor type 4 (CXCR4, PDB: 3ODU, **c**), and endothelin B receptor (ETBR, PDB: 5GLH, **d**).

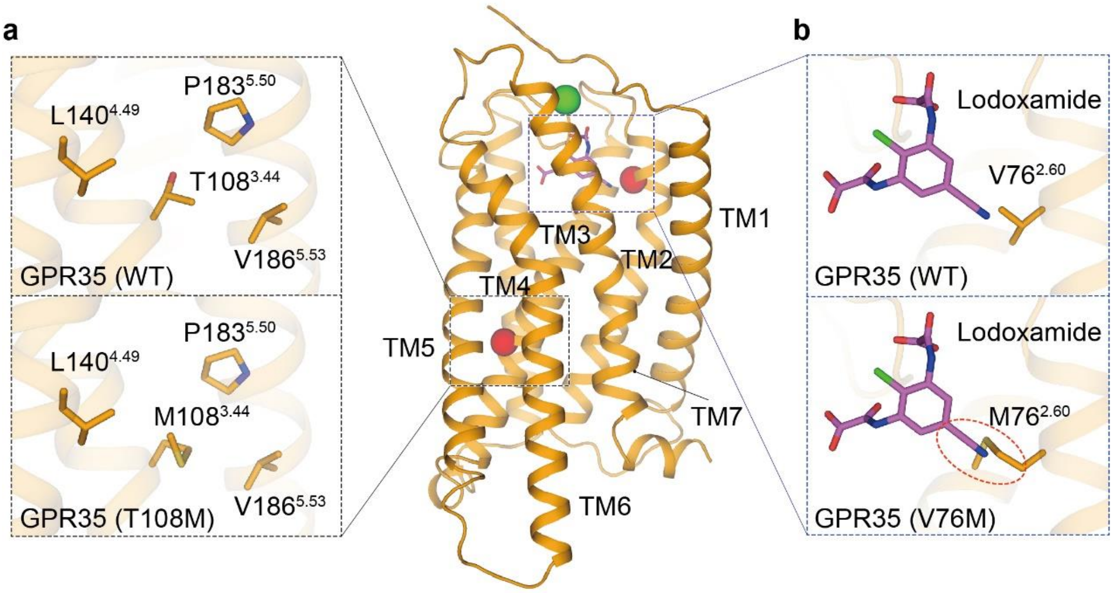

Fig. S6 Disease-associated mutations on GPR35.

Two disease-associated mutations T108$^{3.44}$M (**a**) and V76$^{2.60}$M (**b**) are shown as two red spheres (middle panel). Upper panel, WT GPR35; bottom panel, two disease-associated GPR35 mutants. The potential steric hindrance between lodoxamide and M76$^{2.60}$ is highlighted in a red circle.

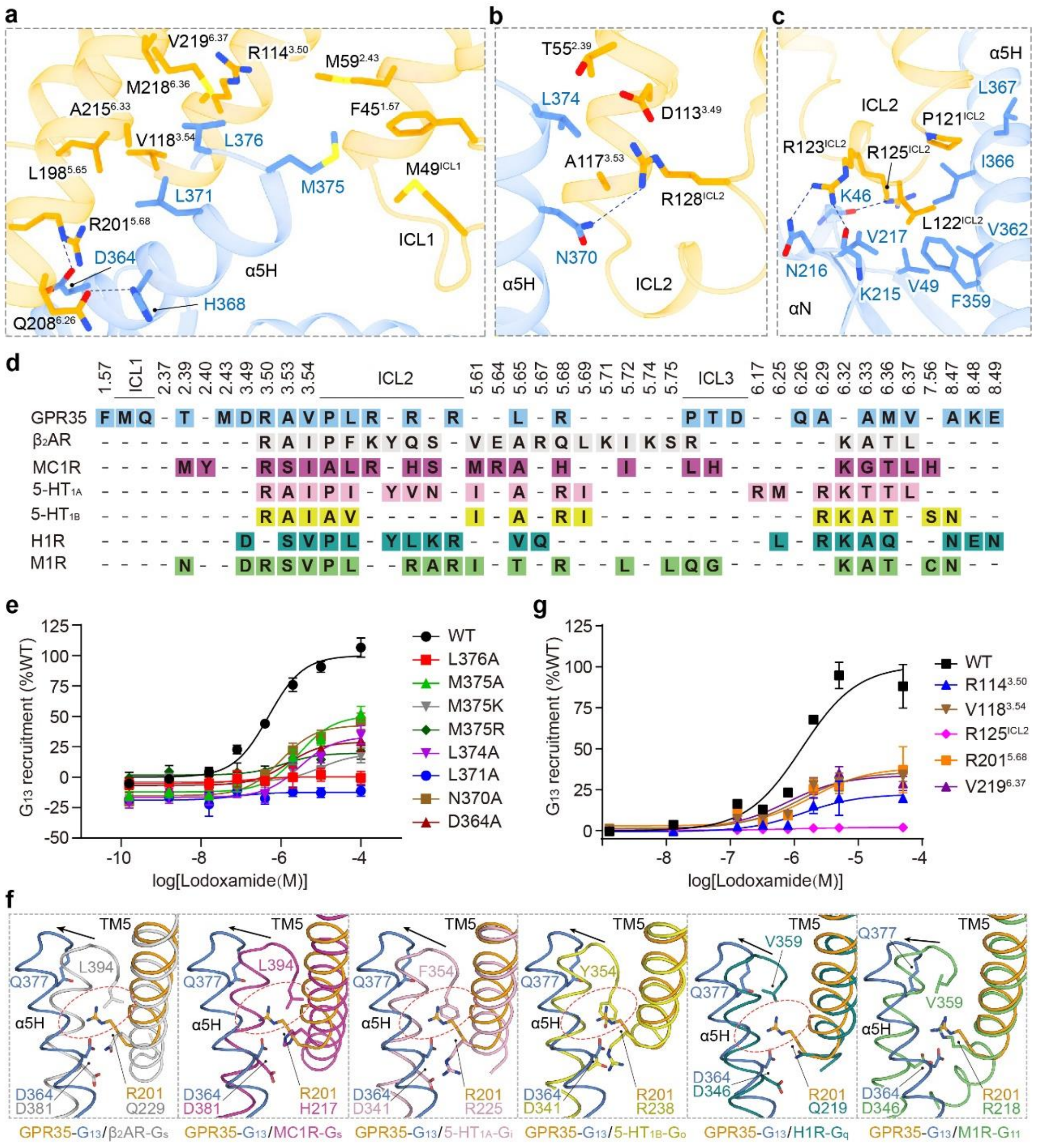

Fig. S7 G_{13}-coupling of GPR35.

a-c Detail interactions between the cytoplasmic cavity of receptor helices and α5 helix of the $G\alpha_{13}$ subunit (**a, b**), and a hydrophobic interface between ICL2 of the receptor and αN and α5 of the $G\alpha_{13}$ subunit (**c**). **d** Comparison of the GPR35-G_{13} coupling profile with that of GPCRs coupled to other G protein subtypes. **e** Concentration-response curves of lodoxamide on $G\alpha_{13}$ mutants at GPR35-G_{13} interfaces. **f** The interactions between D (-14, α5 helix numbering start with -1 from the terminal residue) of α5 helix of Gα subunits and R/Q/H at position 5.68 of receptors. The potential steric hindrance between R/Q/$H^{5.68}$ and the C-terminal residue (-1) and entire α5 helices are highlighted in red dashed circles. The movement of the C-terminus of α5 helix of Gα subunit for GPR35-G_{13} complex compared to other GPCR-G protein complexes are shown as black arrows. **g** Concentration-response curves of lodoxamide on GPR35 mutants at GPR35-G_{13} interfaces.

Table S1 Cryo-EM data collection, model refinement, and validation statistics

	Lodoxamide-GPR35-G_{13}-scFv16 complex
**Data collection and processing**	
Magnification	81,000
Voltage (kV)	300
Electron exposure (e^{-}/Å^2)	50
Defocus range (μm)	-1.0~-2.0
Pixel size (Å)	1.04
Symmetry imposed	C1
Initial particle projections (no.)	32,292,277
Final particle projections (no.)	591246
Map resolution (Å)	3.20
Map resolution range (Å)	2.7-4.0
FSC threshold	0.143
**Model Refinement**	
Refinement package	PHENIX-1.17.1-3660
Real or reciprocal space	Real space
Model-Map CC (mask)	0.74
Model resolution (Å)	3.3
FSC threshold	0.5
B factors (Å2, mean value)	
Protein residues	65.2
Ligands	89.06
**Model composition**	
Non-hydrogen atoms	8,452
Protein residues	1,138
R.m.s. deviations	
Bond lengths (Å)	0.002 (0)
Bond angles (°)	0.522(4)
**Validation**	
MolProbity score	1.58
Clashscore	9.63
Rotamer outliers (%)	0.31
Ramachandran plot	
Favored (%)	97.68
Allowed (%)	2.32
Disallowed (%)	0
**Data availability**	
EMDB entry	EMD-34549
PDB entry	8H8J

Table S2 Allosteric effects of divalent cations on ligands for GPR35 mutants. NanoBiT assay was performed to evaluate the effects of divalent cations on the G protein recruitment of GPR35 and 5-HT_{1A} in the presence of lodoxamide and 5-HT, respectively. The physiological concentrations of Co^{2+}, Zn^{2+}, Cu^{2+}, and Fe^{3+} are much lower than that of Mg^{2+} and Ca^{2+} (WHO Vitamin and Mineral Nutrition Information System, VMNIS). The maximum cell safety concentrations of these cations under our experimental conditions were used. Data are presented as means ± S.E.M. of three independent experiments (n=3). All data were analyzed by two-side, one-way ANOVA with Tukey's test. $^{***}P < 0.001$ *vs.* control.

**Divalent Cations**	**GPR35**		**5-HT$_{1A}$**
	pEC$_{50}$ **(lodoxamide)**	***pEC$_{50}$*** **(zaprinast)**	***pEC$_{50}$*** **(5-HT)**
Control	6.55±0.02	5.88±0.05	7.63±0.02
10 mM Mg^{2+}	$6.99±0.05^{***}$	/	/
100 mM Mg^{2+}	$7.09±0.12^{***}$	$6.44±0.03^{**}$	7.39±0.12
10 mM Ca^{2+}	$7.22±0.15^{***}$	/	/
100 mM Ca^{2+}	$7.16±0.16^{***}$	$7.53±0.18^{***}$	7.71±0.18
1 mM Mn^{2+}	$6.94±0.09^{***}$	/	/
10 mM Mn^{2+}	$7.44±0.03^{***}$	$6.56±0.04^{**}$	7.62±0.11
1 μM Co^{2+}	6.55±0.02	/	/
1 μM Zn^{2+}	6.46±0.04	/	/
0.1 μM Cu^{2+}	6.48±0.05	/	/
10 mM K^{+}	6.52±0.03	/	/
100 mM K^{+}	6.55±0.02	/	/
10 mM Li^{+}	6.53±0.04	/	/
100 mM Li^{+}	6.59±0.07	/	/
100 μM Fe^{3+}	6.56±0.08	/	/

Table S3 Effects of lodoxamide on GPR35 mutants. NanoBiT assay was performed to evaluate the effects of lodoxamide on β-arrestin 2 or G protein recruitment of GPR35. The surface expression of each GPR35 mutant was normalized to wild-type (WT) receptor, which was set to 100%. Data are presented as means ± S.E.M. of three independent experiments (n=3). All data were analyzed by two-side, one-way ANOVA with Tukey's test. $^{*}P$ <0.05, $^{**}P$< 0.01, $^{***}P$ < 0.001, *vs.* WT receptor. U.D., undetectable.

**GPR35 Mutants**	**G protein recruitment**	**Surface Expression (%WT)**
	***pEC_{50}* (lodoxamide)**	
WT	6.02 ± 0.04	100
V76A	< 5.00 ± 0.00	50.57 ± 1.91
L80A	5.07 ± 0.16**	75.15 ± 3.62
Y96A	U.D.	65.52 ± 5.16
R100A	U.D.	147.03 ± 12.54
F163A	U.D.	154.82 ± 8.23
R240A	5.15 ± 0.18**	122.99 ± 2.42
F45A/M49A/M59A	U.D.	164.90 ± 8.11
R114A	6.03 ± 0.14	77.12 ± 4.12
V118A	5.74 ± 0.13	89.74 ± 2.00
R125A	U.D.	111.26 ± 6.30
R201A	5.74 ± 0.28*	76.86 ± 3.59
V219A	6.06 ± 0.08	118.25 ± 4.94
S265G	U.D.	170.67 ± 3.20
S265F	U.D.	169.29 ± 7.48

Table S4 Effects of lodoxamide on Gα_{13} mutants. NanoBiT assay was performed to evaluate the effects of lodoxamide on Gα_{13} mutants recruitment by GPR35. Data are presented as means ± S.E.M. of three independent experiments (n=3). All data were analyzed by two-side, one-way ANOVA with Tukey's test. $^{*}P < 0.05$, $^{**}P < 0.01$, $^{***}P < 0.001$, *vs.* WT Gα_{13} subunit. U.D., undetectable.

**Gα_{13} Mutants**	***pEC$_{50}$***
WT	6.36 ± 0.03
D364A	6.03 ± 0.10*
N370A	5.97 ± 0.07*
L371A	U.D.
L374A	5.67 ± 0.08***
M375A	5.66 ± 0.06***
M375K	5.44 ± 0.22***
M375R	5.79 ± 0.13**
L376A	U.D.
